# Associations between body weight trajectories and neurodevelopment outcomes at 24 months corrected age in very-low-birth-weight preterm infants: a group-based trajectory modelling study

**DOI:** 10.3389/fped.2024.1393547

**Published:** 2024-07-25

**Authors:** Ts-Ting Wang, Yen-Ju Chen, Yi-Han Su, Yun-Hsiang Yang, Wei-Ying Chu, Wei-Ting Lin, Yu-Shan Chang, Yung-Chieh Lin, Chyi-Her Lin, Yuh-Jyh Lin, Taiwan Premature Infant Follow-up Network

**Affiliations:** ^1^ Department of Pediatrics, Ditmanson Medical Foundation Chia-Yi Christian Hospital, Chia-Yi, Taiwan; ^2^ Department of Pediatrics, National Cheng Kung University Hospital, College of Medicine, National Cheng Kung University, Tainan, Taiwan; ^3^ Department of Emergency Medicine, Chi Mei Medical Center, Tainan, Taiwan; ^4^ Department of Pediatrics, E-Da Hospital, Kaohsiung, Taiwan

**Keywords:** neurodevelopment outcome, very-low-birth-weight preterm infants, growth trajectory, extrauterine growth retardation, neurodevelopmental impairment, group-based trajectory modelling

## Abstract

**Introduction:**

This study aimed to explore the relationship between the trajectories of body weight (BW) *z*-scores at birth, discharge, and 6 months corrected age (CA) and neurodevelopmental outcomes at 24 months CA.

**Methods:**

Conducted as a population-based retrospective cohort study across 21 hospitals in Taiwan, we recruited 3,334 very-low-birth-weight (VLBW) infants born between 2012 and 2017 at 23–32 weeks of gestation. Neurodevelopmental outcomes were assessed at 24 months CA. Instances of neurodevelopmental impairment (NDI) were defined by the presence of at least one of the following criteria: cerebral palsy, severe hearing loss, profound vision impairment, or cognitive impairment. Group-based trajectory modeling was employed to identify distinct BW z-score trajectory groups. Multivariable logistic regression was used to assess the associations between these trajectories, postnatal comorbidity, and neurodevelopmental impairments.

**Results:**

The analysis identified three distinct trajectory groups: high-climbing, mid-declining, and low-declining. Significant associations were found between neurodevelopmental impairments and both cystic periventricular leukomalacia (cPVL) [with an adjusted odds ratio (aOR) of 3.59; *p* < 0.001] and belonging to the low-declining group (aOR: 2.59; *p* < 0.001).

**Discussion:**

The study demonstrated that a low-declining pattern in body weight trajectory from birth to 6 months CA, along with cPVL, was associated with neurodevelopmental impairments at 24 months CA. These findings highlight the importance of early weight trajectory and specific health conditions in predicting later neurodevelopmental outcomes in VLBW infants.

## 1 Introduction

NDI is a major concern in preterm population (1, 2), ranging from mild to severe, with long-term adverse outcomes. NDI can affect children's cognitive, motor, language, visual, hearing, and behavioural functioning (3); significantly reduce their quality of life; and impair their academic and social functioning. The causes of NDI are complex and multifactorial. Prematurity is one of the most important risk factors. Other known risk factors include male sex (4); lower parental education level; lower birth weight or gestational age (5); central nervous system conditions such as severe intraventricular haemorrhage (IVH) and cPVL; and respiratory distress syndrome (RDS), bronchopulmonary dysplasia (BPD), sepsis, patent ductus arteriosus (PDA), and advanced retinopathy of prematurity (ROP) (1, 3, 6, 7).

Brain development is sensitive to nutrition status, especially in infancy (8). Adequate brain growth and maturation are positively associated with appropriate BW gain (9, 10). Consequently, growth restriction during the neonatal period is associated with elevated risks of adverse neurodevelopmental outcomes in premature infants (2, 4, 11–15). Regarding the associations between BW and neurodevelopmental outcomes, a study found that an BW *z*-score decrease of more than 1 and 2 standard deviations and a slow weight gain velocity during hospitalisation were associated with NDI incidence at 24 months CA (4). Low BW at a single time point (6, 12, or 24 months CA) may be associated with unfavourable neurodevelopmental outcomes in the VLBW population (12). However, isolated weight assessments, especially for infants below certain weight percentiles, might not offer a comprehensive overview (16). This discrepancy arises because weight measured at specific time points shows weak correlations with longitudinal weight change trends and subsequent neurodevelopmental outcomes (17).

A research gap exists regarding the investigation of the relationship between the longitudinal trajectory assessment of BW increases from birth to the early post discharge period and neurodevelopmental outcomes at 24 months CA. We hypothesised that the patterns of postnatal BW trajectory and neonatal comorbidities was associated with neurodevelopmental outcomes. This study investigated the relationship between the *z*-score trajectory of BW from birth, discharge, to 6 months CA and neurodevelopment outcomes at 24 months CA.

## 2 Materials and methods

### 2.1 Study design

This retrospective cohort study used data from the Taiwan Premature Infant Developmental Collaborative Study Group, which was funded by the Premature Baby Foundation of Taiwan and is collecting follow up data of VLBW infants from 21 hospitals in Taiwan. Infants born between 2012 and 2017 with a birth BW (BBW) <1,500 g, and gestational age (GA) ranged from 23 to 32 weeks were included in this study.

Anthropometric measurements were performed at birth, discharge, and 6 months CA. Neurodevelopmental assessments were performed at 24 months CA. Infants with major anomalies were excluded. To perform group-based trajectory modeling (GBTM), which requires at least three time points of body weight measurements, we excluded patients who died before discharge and those without a BBW record. Patients discharged at a postmenstrual age (PMA) of over 50 weeks (due to the limitations of the Fenton growth chart) were also excluded. During data consolidation, we found that some hospitals had an unusually high proportion of data anomalies for certain years submitted to the Premature Baby Foundation of Taiwan. To enhance the accuracy of our statistics, we removed data from these hospitals (categorized as incomplete data). To improve the precision of follow-up data, we excluded patients who had their follow-ups conducted too early or too late relative to the scheduled age (categorized as follow-up time not within 2 months of the scheduled day). Patients who did not have BSID-III scores at 24 months CA, whether due to death, loss of records from transferring hospitals, or other reasons for loss to follow-up, were also excluded (see online Supplementary Figure S1). This study included 3,334 VLBW newborns. The Institutional Review Board of National Cheng Kung University Hospital approved this study (approval number: ER-109-288).

### 2.2 Variable definitions and outcomes

The collected demographic data included GA, BBW, gender, neonatal morbidities, and post-discharge follow-up information. Anthropometric measurements included BW, body length (BL), and head circumference (HC) at each time point. BW *z*-scores at birth and discharge were determined by the Fenton growth chart (18), while using the World Health Organization standards for growth measurement at 6 months CA. Risk factors included surfactant-treated RDS, severe IVH, PDA requiring treatment, necrotising enterocolitis (NEC) advanced beyond stage 2, ROP advanced beyond stage 3, BPD, and cPVL. Newborns with respiratory distress syndrome caused by hyaline membrane disease, who require support with a fraction of inspired oxygen above 40%, will be treated with surfactant within 48 h after birth. IVH was graded by the Papile classification, with grades 3 and 4 defined as severe. PDA requiring treatment was defined by hemodynamically significant PDA which was treated by surgical or medical intervention. NEC was defined by modified Bell's staging criteria. The stage of ROP is diagnosed based on the criteria established by the International Committee for the Classification of Retinopathy of Prematurity. BPD was defined according to the 2001 National Institute of Child Health and Human Development criteria (19). cPVL was diagnosed by the neurologist through cranial ultrasound, which brain injury involving periventricular white matter.

### 2.3 Neurodevelopment assessment

A child was considered to have NDI at 24 months CA if at least one of the following criteria was met: cerebral palsy, profound vision impairment, severe hearing loss, or cognitive impairment (cognitive composite score of <85) (20). The BSID-III (21) was used to assess neurodevelopment at 24 months CA, including cognitive composite scores. Any of the following symptoms was regarded as a marker of cerebral palsy: hypotonia, spastic diplegia, spastic tetraparesis, or spastic hemiparesis. Profound vision impairment was defined as amblyopia or blindness in both eyes, and severe hearing loss was defined as hearing loss of less than 60 dB in any ear.

### 2.4 Statistical analysis

Group-based trajectory modelling (GBTM) identified clusters of BW *z*-scores at birth, discharge, and 6 months CA. Bayesian information criteria (BIC) determined the optimal cluster count and fittest trajectory shape. Then use average of the posterior probabilities of group membership (APP) to check the modelled trajectories group individuals with similar patterns (22). Parental attributes, neonatal demographics, and postnatal morbidities were compared across three trajectory patterns by using the chi-square or Fisher exact test for categorical variables and the analysis of variance (ANOVA) or Kruskal–Wallis test for continuous variables. Following ANOVA, various trajectory intergroup comparisons were conducted using the Bonferroni *post hoc* test. The logistic regression model was used to identify the variables associated with a risk of NDI at 24 months CA. after a univariate analysis, covariates were selected on the basis of their clinical relevance and between-variable collinearity. Statistical significance was set at *p* < 0.05, and all analyses were performed using R-4.0.2, SPSS (Version 29, IBM, Armonk, NY, USA), and SAS (version 9.4, SAS Institute Inc., Cary, NC, USA) software package with accompanying PROC TRAJ application.

## 3 Results

### 3.1 Study population and patient characteristics

From the original pool of 6,535 newborns that fit our study criteria, a series of exclusions narrowed down our final analysis to 3,334 infants (online Supplementary Figure S1). Infants with major anomalies (*n* = 84), with mortality before discharge (*n* = 730), no BBW records (*n* = 1), who were discharged at a PMA of >50 weeks (*n* = 119), follow up timing beyond 2 months than the regular schedule (*n* = 749), and who did not have data available on BSID scores at 24 months CA (*n* = 1,068) were excluded. The reasons for the lack of data on BSID scores at 24 months CA encompassed transfer to another hospital (*n* = 36) and mortality after discharge but before reaching 24 months CA (*n* = 26). In addition, one hospital did not provide complete annual data to the foundation because of concerns regarding data integrity and accuracy; therefore, infants from this hospital were excluded from our analysis (*n* = 450). The average gestational age was 28.45 weeks (28.45 ± 2.26), 50.87% of newborns were male, and 14.97% were small for gestational age. During the neonatal period, 33.62% were diagnosed with RDS and treated with surfactant, 3.57% had severe IVH, 23.94% had PDA and required medication or surgical treatment, 5.01% were diagnosed as having NEC, 24.75% had severe ROP, 37.31% had BPD, and 4.05% had cPVL. At 24 months CA, the mean cognitive, language, and motor composite score were 94.35 (94.35 ± 12.63), 92.29 (92.29 ± 13.79), and 91.74 (91.74 ± 12.41), respectively. NDI was diagnosed in 23.94% of infants (Table 1).

**Table 1:** Demographic characteristics of the cohort.

Variable	*N* = 3,334	Missing data	Range
Parental characteristics			
Maternal education <high school, %	1,564 (46.8)	63	
Paternal education <high school, %	1,439 (43.1)	296	
Multiple pregnancy, %	973 (29.2)	2	
Neonatal characteristics			
GA, weeks, mean ± SD	28.5 ± 2.3	0	23–32
Gender (male), %	1,696 (50.9)	0	
BBW, gram, mean ± SD	1,095 ± 259.2	0	407–1,500
BBL, cm, mean ± SD	36.3 ± 3.5	31	22.8–49
BHC, cm, mean ± SD	25.7 ± 2.2	43	17–33
SGA, %	499 (15)	0	
Discharge BW, gram, mean ± SD	2,572 ± 556.7	25	1,658–7,856
Discharge BL, cm, mean ± SD	45.6 ± 3.1	572	30.5–62
Discharge HC, cm, mean ± SD	32.1 ± 1.8	626	23–40
Length of stay at NICU, mean ± SD	57.5 ± 30.3	3	2–175
Discharge PMA, mean ± SD	38.4 ± 2.8	0	22.8–49.8
Postnatal comorbidity			
Surfactant treated RDS, %	1,121 (33.6)	164	
Severe IVH (≥grade 3),%	119 (3.6)	0	
PDA with treatment, %	798 (23.9)	5	
NEC stage ≥2, %	167 (5)	0	
ROP stage ≥3, %	825 (24.8)	34	
BPD, %	1,244 (37.3)	0	
cPVL, %	135 (4.1)	0	
Neurodevelopment at 24 months CA			
Cognitive composite score, mean ± SD	94.4 ± 12.6	3	10–145
Language composite score, mean ± SD	92.3 ± 13.8	3	47–147
Motor composite score, mean ± SD	91.7 ± 12.4	3	33–136
Profound hearing loss, %	25 (0.75)	868	
Cerebral palsy, %	99 (3)	70	
Bilateral blindness, %	2 (0.1)	139	
Neurodevelopmental impairment, %	798 (23.9)	0	

GA, gestational age; SD, standard deviation; BBW, birth body weight; BBL, birth body length; BHC, birth head circumference; SGA, small for gestational age; BW, body weight; BL, body length; HC, head circumference; RDS, respiratory distress syndrome; IVH, intraventricular hemorrhage; PDA, patent ductus arteriosus; NEC, necrotizing enterocolitis; ROP, retinopathy of prematurity; BPD, bronchopulmonary dysplasia; cPVL, cystic periventricular leukomalacia; CA, corrected age; NICU, neonatal intensive care unit; PMA, postmenstrual age.

### 3.2 BW *z*-score trajectory

**Figure 1:**
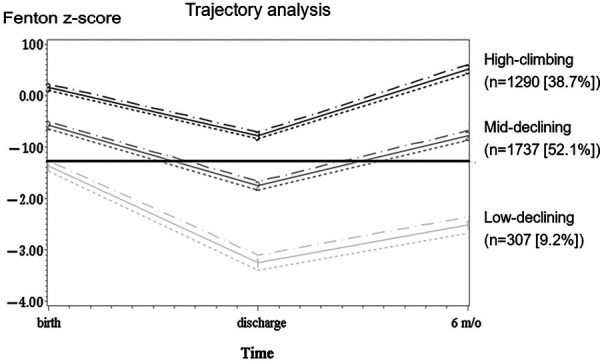
Trajectory patterns of body weight *z*-scores. The trajectory patterns of body weight *z*-scores from birth to 6 months corrected age. The body weight *z*-score trajectory was classified into three trajectory groups: low-declining group [*n* = 307 (9.2%)], mid-declining group [*n* = 1,737 (52.1%)], and high-climbing group [*n* = 1,290 (38.7%)]. The dotted lines represent 95% confidence intervals.

Using GBTM, based on BW *z*-scores at birth, discharge, and 6 months CA, we found three quadratic shape trajectory group has the highest BIC. Based on the characteristics of the trajectories, the three trajectory groups were named high-climbing, mid-declining, and low-declining groups (Figure 1). These groups comprised 307 (9.2%), 1,737 (52.1%), and 1,290 (38%) of the infants, respectively. On average, the posterior probabilities of group membership were all >0.7 (0.86, 0.84, 0.85, respectively). The odds of correct classification for the three groups were 62.11, 4.85, and 8.86, respectively (online Supplementary Table S1). All trajectories had a *z*-score nadir upon discharge; thereafter, the *z*-score improved at 6 months CA. From a trend perspective, the high-climbing group demonstrates that their BW *z*-score at 6 months CA surpasses their BBW *z*-score. In contrast, the other two groups do not exhibit this pattern. Notably, the low-declining group shows a BW *z*-score at 6 months CA that is significantly lower than their BBW *z*-score. The characteristics and neonatal comorbidities of the three trajectory groups are shown in Table 2. Significant differences were observed in GA, *z*-scores for anthropometric measurements taken at birth, incidence of small for gestational age (SGA), PDA requiring treatment, NEC, severe ROP, BPD, and cPVL (Table 2).

**Table 2:** Differences in parental and neonatal characteristics and neonatal comorbidities among the 3 body weight growth trajectory groups.

	Low declining (*N* = 307)	Mid declining (*N* = 1,737)	High climbing (*N* = 1,290)	*p*-value
	*n* (%)/ mean ± SD	*n* (%)/ mean ± SD	*n* (%)/ mean ± SD	
Parental characteristics				
Maternal education ≤ high school	131 (43.52)	816 (47.83)	614 (48.58)	0.286
Paternal education ≤ high school	121 (44.81)	734 (46.11)	584 (49.66)	0.122
Multiple pregnancy	84 (27.36)	527 (30.3)	362 (28.06)	<0.001*
Neonatal characteristics				
GA	28.71 ± 2.52	28.67 ± 2.44	28.09 ± 1.86	<0.001*
Gender	188 (61.24)	881 (50.7)	627 (48.6)	0.298
BBW z score	−1.42 ± 0.74	−0.61 ± 0.68	0.22 ± 0.62	<0.001*
BBL z score	−1.76 ± 1.13	−0.66 ± 0.99	0.19 ± 0.96	<0.001*
BHG z score	−1.59 ± 1.19	−0.61 ± 0.97	0.15 ± 0.93	<0.001*
SGA	201 (65.47)	296 (17.04)	2 (0.16)	<0.001*
Postnatal comorbidity				
Surfactant-treated RDS	120 (39.09)	573 (34.79)	428 (34.80)	0.115
Severe IVH (≥grade 3)	16 (5.21)	68 (3.91)	35 (2.71)	0.056
PDA with treatment	93 (30.29)	425 (24.47)	280 (21.71)	0.005*
NEC stage ≥ 2	31 (10.10)	97 (5.58)	39 (3.02)	<0.001*
ROP stage ≥ 3	98 (31.92)	457 (26.60)	270 (21.14)	<0.001*
BPD	171 (55.70)	638 (36.73)	435 (33.72)	<0.001*
cPVL	20 (6.51)	65 (3.74)	50 (3.88)	0.070

SD, standard deviation; GA, gestational age; BBW, birth body weight; BBL, birth body length; BHG, birth head circumference; SGA, small for gestational age; RDS, respiratory distress syndrome; IVH, intraventricular haemorrhage; PDA, patent ductus arteriosus; NEC, necrotising enterocolitis; ROP, retinopathy of prematurity; BPD, bronchopulmonary dysplasia; cPVL, cystic periventricular leukomalacia.

* Values marked in bold indicate statistical significance (*P* < 0.05).

### 3.3 Association between BW *z*-score trajectory and NDI

The study used logistic regression to analyse variables, such as BW *z*-score trajectory, maternal/paternal education level, gender, GA, BBW/BBL/BHC *z*-score, SGA, surfactant-treated RDS, severe IVH, PDA requiring treatment, NEC advanced beyond stage 2, ROP advanced beyond stage 3, BPD, and cPVL, to determine whether they were significantly associated with NDI at 24 months CA. According to a univariate analysis, there was no significant correlation between the maternal/paternal education level, BBW/BBL/BHCṭ *z*-score, SGA, and NDI at 24 months CA (*p* = 0.79, 0.86, 0.56, 0.59, 0.1, 0.28, respectively; online Supplementary Table S2). A multivariate analysis demonstrated that neonatal morbidities, including surfactant-treated RDS, severe IVH, and ROP stage 3, were positively correlated with NDI. However, the cPVL and low-declining groups had the highest aOR associated with NDI at 24 months CA (aOR: 3.59; 95% CI: 2.47–5.24; *p* < 0.001) and low-declining group (aOR: 2.59; 95% CI: 1.92–3.48; *p* < 0.001) (Table 3).

**Table 3:** Crude and adjusted odds ratios for neurodevelopment impairment at 24 months corrected age stratified by risk factors and *z*-score body weight trajectory.

	Univariate analysis		Multivariable analysis	
	Crude OR (95% CI)	*p*-value	Adjusted OR (95% CI)	*p*-value
Trajectory groups
Low declining	2.68 (2.05–3.50)	<0.001	2.59 (1.92–3.48)	**<0** **.** **001** *
Mid declining	1.33 (1.12–1.59)	0.001	1.37 (1.13–1.65)	**0**.001*
High climbing	Ref.		Ref.	
Maternal education ≤ high school	1.02 (0.87–1.20)	0.790		
Surfactant treated respiratory distress syndrome	2.04 (1.73–2.41)	<0.001	1.33 (1.09–1.61)	**0**.005*
Severe Intraventricular haemorrhage	3.41 (2.36–4.93)	<0.001	1.78 (1.19–2.66)	**0**.005*
Patent ductus arteriosus with treatment	1.61 (1.35–1.92)	<0.001	1.08 (0.88–1.32)	0.461
Necrotizing enterocolitis stage ≥ 2	1.65 (1.18–2.30)	0.003	1.27 (0.89–1.81)	0.187
Retinopathy of prematurity stage ≥ 3	1.91 (1.60–2.27)	<0.001	1.34 (1.10–1.63)	**0**.004*
Bronchopulmonary dysplasia	1.78 (1.52–2.10)	<0.001	0.98 (0.80–1.20)	0.861
Cystic periventricular leukomalacia	4.71 (3.32–6.70)	<0.001	3.59 (2.47–5.24)	**<0**.001*

Adjusted for gestational age and gender.

* Values marked in bold indicate statistical significance (*P* < 0.05).

## 4 Discussion

The definition of extrauterine growth restriction (EUGR) is challenged by the arbitrary cut-off value set at either 36 weeks postmenstrual age or at the time of discharge. This definition may not be useful as weight at a specific time point shows weaker correlations with long-term weight change trends and future neurodevelopment compared to continuous observations (17). Some scholars seem to agree that EUGR associated with poor neurodevelopment outcome. However, there continues to be debate about whether EUGR is predictive of poor neurodevelopment (23, 24). Therefore, different evaluation methods, such as different growth charts for measuring BW (11), BW measurement at a single time point (11, 12, 24), BW *z*-score differences in each period, or growth velocity (4, 13, 25, 26), were used to analyse the relationship between growth status and neurodevelopment outcomes. In this nationwide retrospective cohort study, we examined the growth trajectory at birth, discharge, and 6 months CA through GBTM. We observed that the weight *z*-scores of the three patient groups all decreased to their lowest point at discharge after the application of GBTM, followed by an increase at 6 months CA. The low-declining group comprises 65.47% of the SGA population, as indicated by Table 2 and Figure 1. In contrast, the high-climbing group, which began with a *z*-score of >0, has 0.16% of SGA individuals. The high-climbing group surpasses their birth weight *z*-score by 6 months CA, whereas the low-declining group remains significantly below their birth weight *z*-score. Our logistic regression univariate analysis, which combined birth weight *z*-score and SGA status (Supplementary Table S2), did not reveal a significant association between NDI and either variable (*p* = 0.562 and *p* = 0.277, respectively). Our findings indicated that the probability of NDI can be ascertained by analysing long-term weight trends. The NDI at CA 24 months exhibits a substantial correlation with the growth trajectory, particularly the capacity to catch up by CA 6 months, in contrast to the birth weight *z*-score or SGA status at birth. Poor growth may result from various complications in the early life of preterm infants, ultimately leading to NDI. Therefore, when assessing NDI, various risk factors should be considered (2, 7). Our results indicated that infants with cPVL, severe IVH, RDS requiring surfactant therapy or those with extensive ROP are susceptible to NDI. cPVL and severe IVH are well-documented for their adverse neurological impacts (27, 28), Our findings align with previous research, demonstrating that both cPVL and the low-declining growth trajectory were significant associated with NDI at 24 months CA. Early RDS and advanced ROP are indicators of postnatal growth limitations (29). In addition, research has demonstrated that low parental education, PDA requiring treatment, and NEC are risk factors for poor neurodevelopmental outcomes (1, 6, 30) and may be associated with IVH (11, 31). However, our investigation revealed no correlation between these variables. The reason for the discrepancy between these studies and our findings is unknown, but it may be due to aggressive treatment strategies for PDA in some hospitals, which may prevent patients with hemodynamically significant PDA from experiencing adverse effects on brain development and a weak association between PDA and NDI. Although previous studies have reported an association between NDI and BPD (32–34), we did not find a robust relationship between these conditions. Bauer et al. also found no increase in NDI prevalence among BPD patients (35).

### 4.1 Strength and limitations

The strength of this study lies in its comprehensive multi-center database. Unlike some studies that excluded (6, 11, 12) conditions like high-grade IVH or cPVL due to their adverse neurodevelopmental impacts (27, 28), we included infants with these two comorbidities given their strong correlation with NDI at 24 months CA. Our study may be among the few to evaluate the association between growth and neurodevelopment outcomes through GBTM.

There are limitations in this cohort study. Sepsis in VLBW neonates often leads to worse neurodevelopmental outcomes (36), achieving full oral nutrition by 40 weeks postmenstrual age is associated with improved neurodevelopmental outcomes (37), administration of antenatal steroid and magnesium sulfate are associated with reduced risk of childhood impairment (38), and delay cord clamping in preterm infants is associated with lower incidence of intraventricular hemorrhage (39). Temperature instability upon NICU admission, including both hyperthermia and hypothermia, can impact adverse neurological development. All of these risk factors are linked to NDI, but we couldn't find that information in our database.

In cases where infants were discharged alive, a loss-to-follow-up rate of 24% was observed at 24 months CA, of which only 2% was due to mortality after discharge. In the future, prospective and longitudinal studies should be conducted on this topic.

## 5 Conclusion

The BW trajectory pattern before 6 months CA showed a significant association with NDI at 24 months CA. This association suggests that growth reaching normal levels, which means an appropriate growth pattern, should be emphasised in both hospitalisation and early discharge periods before 6 months CA. More RCT are required to determine whether interventions administered to improve BW trajectory in early infancy can improve neurodevelopmental outcomes later in life.

## Data Availability

The original contributions presented in the study are included in the article/Supplementary Material, further inquiries can be directed to the corresponding author.
