# Supplementary material for: Associations between body weight trajectories and neurodevelopment outcomes at 24 months corrected age in very-low-birth-weight preterm infants: a group-based trajectory modelling study
Source: Front Pediatr. 2024 Jul 25;12:1393547. doi: 10.3389/fped.2024.1393547 (PMC11306191; doi:10.3389/fped.2024.1393547)
Supplement: Supplementary file 1 [file Datasheet1.pdf]

Supplementary Material

Supplementary Figure

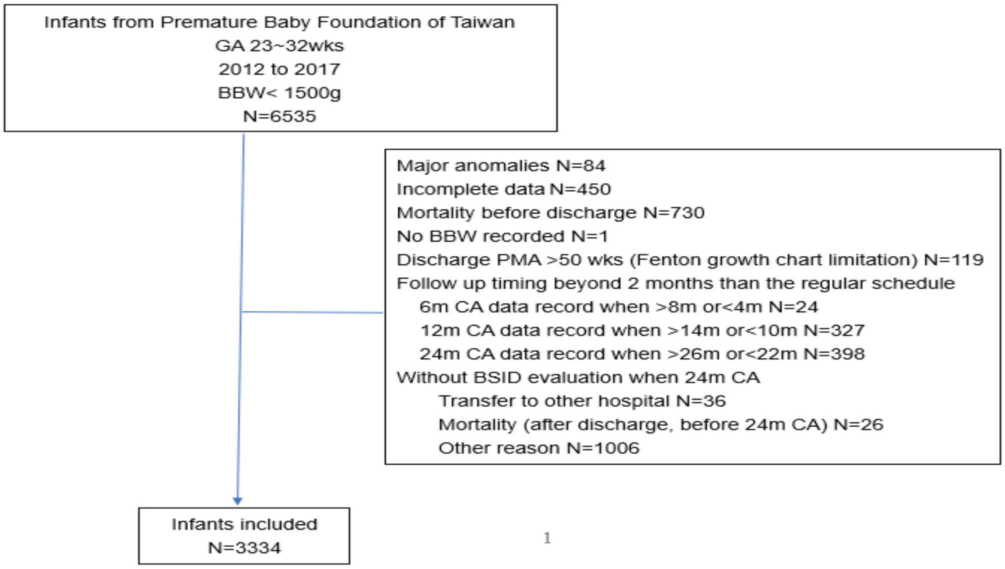

Supplementary Figure 1. Flow diagram depicting the selection of the study population.

SUPPLEMENTAL TABLE 1 Group-based trajectory model diagnostics (three time points).

Model	N	APP	OCCj	Pj	πj	% difference between Pj and πj	BIC
**Three group**							-13444.76
1 (Low-declining group)	307	0.86	62.11	0.10	0.09	11.11	
2 (Mid-declining group)	1737	0.84	4.85	0.51	0.52	1.92	
3 (High-climbing group)	1290	0.85	8.86	0.39	0.39	0.00	
Trajectory 1 (low-declining group) followed a quadratic trend ($\beta0 = 3.11$ [$p < 0.001$]; $\beta1 = -5.79$ [$p < 0.001$]; $\beta2 = 1.30$ [$p < 0.001$]). Trajectory 2 (mid-declining group) followed a quadratic trend ($\beta0 = 2.74$ [$p < 0.001$]; $\beta1 = -4.39$ [$p < 0.001$]; $\beta2 = 1.07$ [$p < 0.001$]). Trajectory 3 (high-climbing group) followed a quadratic trend ($\beta0 = 3.32$ [$p < 0.001$]; $\beta1 = -4.27$ [$p < 0.001$]; $\beta2 = 1.11$ [$p < 0.001$]).							

APP, average of the posterior probabilities of group membership; OCCj. odds of correct classification; Pj, posterior probabilities of group membership; πj, proportion of group membership; BIC, bayesian information criteria

SUPPLEMENTAL TABLE 2 Results of the multivariable ordinal logistic regression analyses for neurodevelopment impairment at 24 months corrected age.

	Univariate analysis		Multivariable analysis	
	**Crude OR (95% CI)**	**p-value**	**Adjusted OR (95% CI)**	**p-value**
Trajectory groups				
Low declining	2.68 (2.05-3.50)	<0.001	2.59(1.92-3.48)	<0.001*
Mid declining	1.33 (1.12-1.59)	0.001	1.37(1.13-1.65)	0.001*
High climbing	Ref.		Ref.	
Gender	0.63(0.54-0.74)	<0.001	0.68(0.58-0.81)	<0.001*
GA	0.83(0.80-0.86)	<0.001	0.88(0.84-0.92)	<0.001*
Maternal education ≤ high school	1.02(0.87-1.20)	0.790		
Paternal education ≤ high school	1.02(0.86-1.20)	0.857		
Surfactant treated RDS	2.04(1.73-2.41)	<0.001	1.33(1.09-1.61)	0.005*
Severe IVH (≥ grade 3)	3.41(2.36-4.93)	<0.001	1.78(1.19-2.66)	0.005*
PDA with treatment	1.61(1.35-1.92)	<0.001	1.08(0.88-1.32)	0.461
NEC stage ≥ 2	1.65(1.18-2.30)	0.003	1.27(0.89-1.81)	0.187
ROP stage ≥ 3	1.91(1.60-2.27)	<0.001	1.34(1.10-1.63)	0.004*
BPD	1.78(1.52-2.10)	<0.001	0.98(0.80-1.20)	0.861
Cystic PVL	4.71(3.32-6.70)	<0.001	3.59(2.47-5.24)	<0.001*

BBW z score	0.97(0.86-1.07)	0.562		
BBL z score	0.98(0.92-1.05)	0.590		
BHG z score	0.94(0.88-1.01)	0.102		
SGA	1.13(0.91-1.40)	0.277		

OR, odds ratio; GA, gestational age; BBW, birth body weight; BBL, birth body length; BHG, birth head circumference; SGA, small for gestational age; RDS, respiratory distress syndrome; IVH, intraventricular haemorrhage; PDA, patent ductus arteriosus; NEC, necrotising enterocolitis; ROP, retinopathy of prematurity; BPD, bronchopulmonary dysplasia; PVL, periventricular leukomalacia.

* Statistically significant at $P < 0.05$.
